# Lessons for the clinical nephrologist: a rare case with MGRS characterized by combined crystalline light chain proximal tubulopathy and crystal-storing histiocytosis responding to daratumumab

**DOI:** 10.1007/s40620-023-01584-1

**Published:** 2023-03-01

**Authors:** Ai-bo Qin, Xiao-juan Yu, Xi-zi Zheng, Su-xia Wang, Fu-de Zhou, Ming-hui Zhao

**Affiliations:** 1 Renal Division, Department of Medicine, Peking University First Hospital, No. 8, Xishiku Street, Xicheng District, Beijing, China; 2 Institute of Nephrology, Peking University, Beijing, China; 3 Renal Pathology Center, Institute of Nephrology, Peking University, Beijing, China; 4 Key Laboratory of Renal Disease, Ministry of Health of China, Beijing, China; 5 Key Laboratory of CKD Prevention and Treatment, Ministry of Education of China, Beijing, China; 6 Research Units of Diagnosis and Treatment of Immune-Mediated Kidney Diseases, Chinese Academy of Medical Sciences, Beijing, China; 7 Laboratory of Electron Microscopy, Pathological Centre, Peking University First Hospital, Beijing, Beijing, 100034 China; 8 Peking-Tsinghua Center for Life Sciences, Beijing, China

**Keywords:** Monoclonal gammopathy, Monoclonal gammopathy of renal significance, Crystal-storing histiocytosis, Light chain proximal tubulopathy, Daratumumab

## The case

A 67-year-old male presented with a 3-year history of proteinuria and hematuria. Baseline serum creatinine (SCr) was 112 μmol/L. Twenty days before admission, he had fever up to 38.6 °C and acute dysuria, after swimming. After taking ibuprofen, azithromycin, and Chinese herbs for 7 days and receiving levofloxacin intravenously for 3 days in a local clinic, his temperature returned to normal. However, he developed dysuria and acute kidney injury (AKI) while SCr rose to 925 μmol/L. Abdominal CT scan revealed urine retention and prostate enlargement. After urethral catheterization, he was transferred to our hospital.

On admission, the patient’s SCr had decreased to 651 μmol/L. He presented with moderate proteinuria (2.01 g/24 h), mild leukocyturia (white blood cells 5–9/HPF), and hypophosphatemia, hypokalemia, and renal glycosuria. Urine protein electrophoresis showed a high proportion of low molecular weight proteinuria (50.1%). A monoclonal free *κ* spike was detected in urine by immunofixation electrophoresis. He had abnormal serum free light chain (sFLC) levels: serum free κ light chain of 325 mg/L, free *λ* light chain of 32.3 mg/L, with a *κ*/*λ* ratio of 10.1, and a difference between involved and uninvolved sFLC (dFLC) of 292.7 mg/L. Bone marrow aspiration revealed 2% plasma cells with 0.2% abnormal plasma cells and κ light chain restriction.

**Fig. 1:**
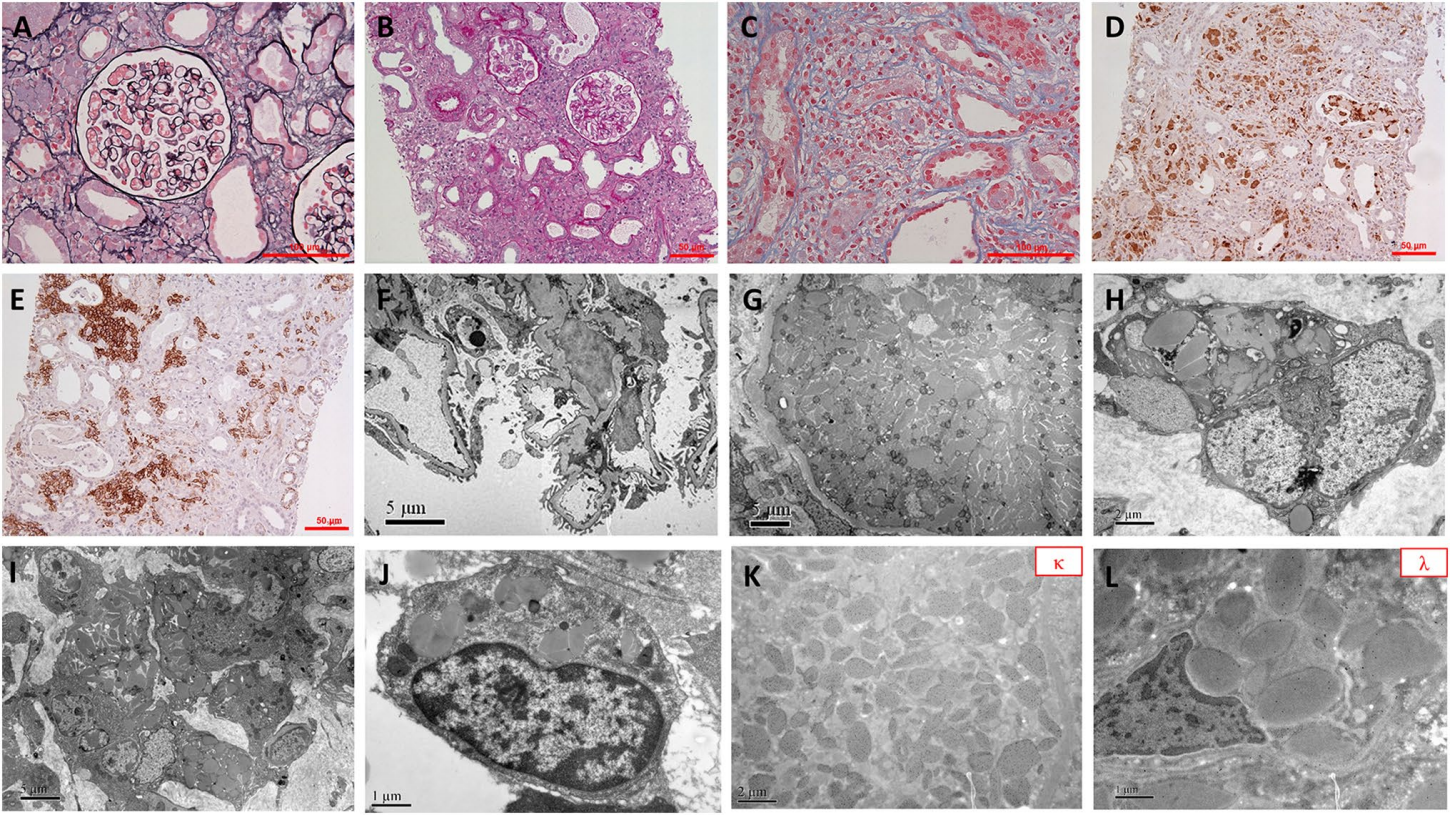
Findings on kidney biopsy. **A** Glomeruli of mild change (PASM + Masson staining, × 200). **B** The figure shows prominent tubulointerstitial lesions: massive interstitial cell infiltration, desquamation of tubular epithelial cells, and some cell debris in the tubular lumen (PAS staining, × 100). **C** Massive crystal inclusions in the cytoplasm of some tubular epithelial cells (Masson staining, × 200). **D** These inclusion-containing cells were CD68 positive (Immunohistochemistry, × 100). **E** There was massive CD138 positive plasma cell infiltration. (Immunohistochemistry, × 100). **F** Normal glomeruli on electron microscopy without any deposits (EM, × 6000). **G** Different shapes of crystals in the cytoplasm of the proximal tubular epithelial cells (EM, × 4000). **H** Rhomboid-shaped intracytoplasmic crystalline inclusion in histiocytes (EM, × 4000). **I** Rhomboid-shaped intracytoplasmic crystalline inclusion in histiocytes (EM, × 10,000). **J** Rhomboid-shaped crystalline inclusion in the cytoplasm of bone marrow histiocytes (EM, × 20,000). **K** Immuno-electron microscopy showed these crystals were κ light chain positive (EM, × 10,000). **L** Immuno-electron microscopy showed these crystals were λ light chain negative (EM, × 20,000)

A kidney biopsy was performed, with suspicion of monoclonal κ light chain-induced tubulointerstitial injury. Light microscopy examination revealed mild changes in most glomeruli (Fig. 1A). Massive interstitial cell infiltration and desquamation of tubular epithelial cells were observed (Fig. 1B). Crystal inclusions were observed in the cytoplasm of some tubular epithelial cells as well as in the cytoplasm of CD68-positive histiocytes massively infiltrating the interstitium. (Fig. 1C, D). There were massive infiltrates of CD138-positive plasma cells in the renal interstitium with κ light chain restriction (Fig. 1E). Congo red staining for amyloid was negative. Electron microscopy examination revealed no obvious lesions or electron-dense deposits in the glomeruli (Fig. 1F). However, there were massive, variously shaped intracytoplasmic crystalline inclusions in the cytoplasm of the proximal tubular epithelial cells (Fig. 1G), in the cytoplasm of renal interstitial histiocytes (Fig. 1G, I), and in bone marrow histiocytes (Fig. 1J). Immuno-electron microscopy revealed κ light chain in the crystals (Fig. 1K) (Fig. 1L). The patient was diagnosed with monoclonal gammopathy of renal significance (MGRS), which manifested as light chain proximal tubulopathy (LCPT) combined with crystal-storing histiocytosis (CSH).

**Fig. 2:**
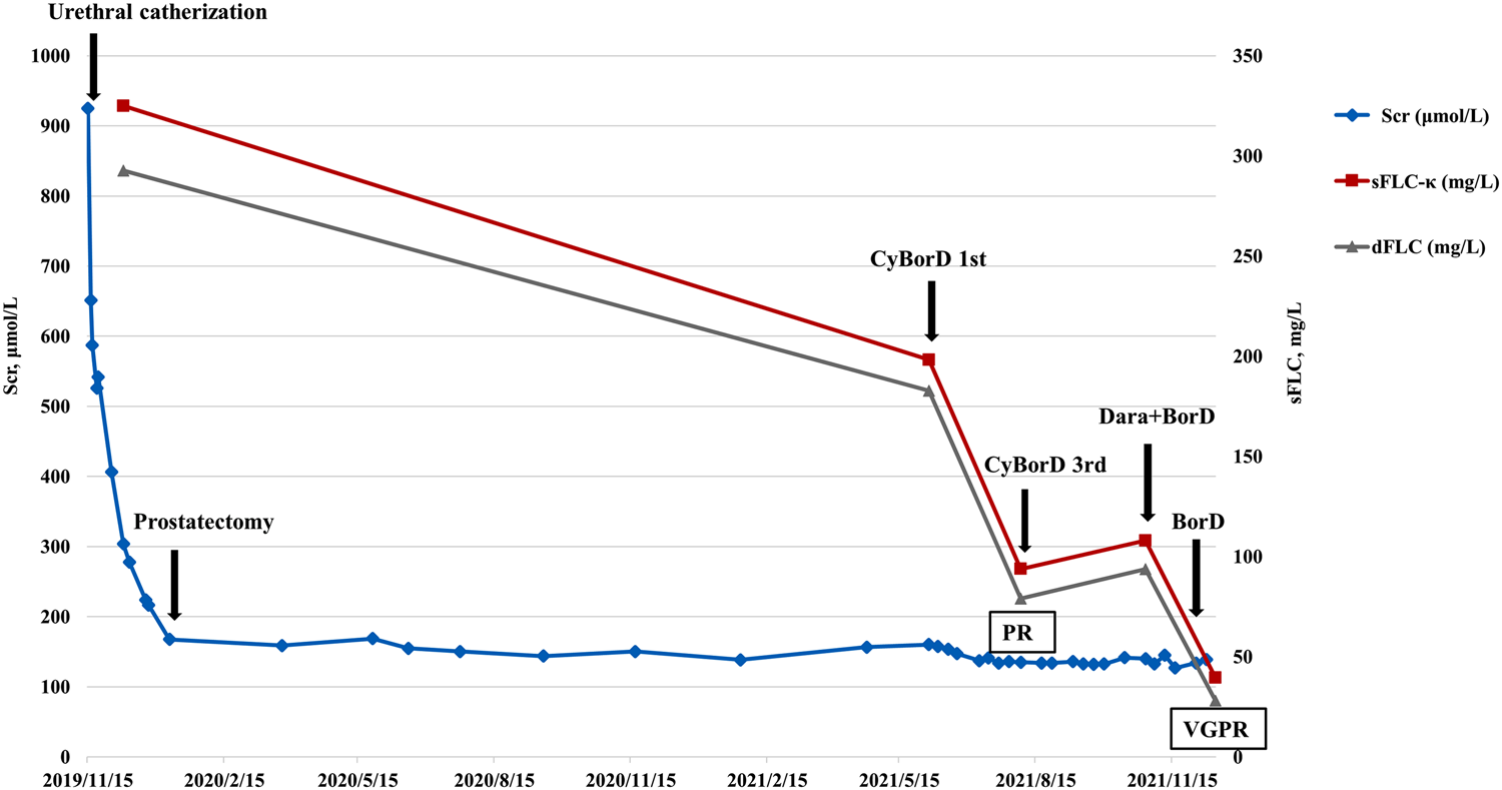
Therapeutic effects over the disease course. *BorD* bortezomib and dexamethasone, *CyBorD* cyclophosphamide, bortezomib, and dexamethasone, *Dara* daratumumab, *sFLC* serum free light chain, *dFLC* the difference between involved and uninvolved sFLC, *sFLC-κ* serum free κ light chain, *SCr* serum creatinine, *PR* partial response, *VGPR* very good partial response

The patient initially refused chemotherapy. He was referred to the urology department for prostatectomy due to the enlarged prostate. Six months after the kidney biopsy, SCr decreased to 160.4 μmol/L, but there was no decrease in proteinuria, and sFLC level was still high. He consented to be treated with bortezomib-based chemotherapy. After two cycles of CyBorD (cyclophosphamide, bortezomib, and dexamethasone) regimen, the dFLC level decreased to 79 mg/L from 292.7 mg/L, and he achieved partial response (PR). However, dFLC levels slightly increased to 93.7 mg/L after the following two cycles of CyBorD, so we administered daratumumab intravenously once a week for four consecutive weeks combined with BorD (bortezomib and dexamethasone) for the fifth cycle. To complete treatment, we administered a further cycle of BorD. The dFLC level decreased to 28.2 mg/L, but monoclonal free *κ* light chain was still detected in his urine, suggesting very good partial response (VGPR). To date, the patient has been followed up for 30 months, and SCr is stable at approximately 125–140 μmol/L (Fig. 2). In addition, his Fanconi syndrome improved.

## Lessons for the clinical nephrologist

Monoclonal gammopathy of renal significance refers to a group of renal disorders caused by monoclonal immunoglobulin (MIg) secreted by a nonmalignant or premalignant B cell or plasma cell clone [1]. MGRS-associated renal lesions encompass a wide spectrum of renal pathology, among which, LCPT and CSH, characterized by monoclonal immunoglobulin accumulation and aggregation as crystals within proximal tubular epithelial cells and histiocytes [2], have seldom been reported. Combined LCPT and CSH is even rarer, with only 11 cases reported in the literature (Table S1, Supplementary Appendix). Furthermore, most of the reported cases were associated with multiple myeloma. We discuss this unusual case of a 67-year-old male with MGRS characterized by combined LCPT and CSH responding to daratumumab.

### Diagnostic clues to this case

This patient presented with moderate proteinuria, partial Fanconi syndrome, and AKI, which improved with supportive treatment. First, the significant reduction in SCr after urethral catheterization indicated that acute obstructive nephropathy due to prostatic hyperplasia was an additional cause of AKI. Drug-induced acute interstitial nephritis was initially suspected due to the medications administered, eosinophilia, and rash. Further laboratory tests revealed monoclonal κ chains in the urine with abnormal serum free light chains. In a study published recently, Klomjit N et al. found that proteinuria ≥ 1.5 g/day, hematuria, and an elevated free light chain ratio increase the likelihood of finding MGRS [3], and a study from our center confirmed that abnormal free light and proteinuria ≥ 1.5 g/day were predictors of MGRS [4]. Kidney biopsy should be highly recommended in such patients. After multiple myeloma and other malignant lymphoplasmacytic proliferative disorders were ruled out, kidney biopsy confirmed the diagnosis of MGRS characterized by combined monoclonal *κ*-restricted CSH and crystalline LCPT.

### The pathogenic mechanism of LCPT and/or CSH

CSH is a rare complication of multiple myeloma and B cell lymphoproliferative disorders, characterized by the accumulation of light chain crystals in histiocytes, primarily in the bone marrow but also in the kidney [5, 6]. The mechanisms of crystallization of immunoglobulins and of their accumulation in various cells are not fully understood. Consistent with the present case, most crystalline immunoglobulin deposits are reported to be caused by monoclonal *κ* light chains [2, 6]. It was suspected that the light chain isotype is a major determinant of crystallization. Researchers suggested that unique mutations in the variable domains of the monoclonal *κ* light chain could result in the substitution of polar residues by hydrophobic residues, inducing the *κ* crystals to be resistant to normal lysosome enzyme proteolysis. Then the undigested light chains form highly organized crystals within the endolysosomal compartment under certain conditions. In the present case, the presence of the *κ* light chain was confirmed by immuno-electron microscopy in both proximal tubular epithelium and interstitial histiocytes. In addition, the crystals in CSH resembled those that presented within proximal tubule cells, suggesting that LCPT and CSH may share a similar pathogenesis.

### How do we treat MGRS-associated LCPT or CSH?

The optimal therapy for patients with MGRS-associated LCPT or CSH is not established. The therapeutic decision should be made depending on the severity of the kidney lesion and the risk of progression. In patients who present with significant kidney injury and/or proteinuria, the International Kidney and Monoclonal Gammopathy Research Group suggests chemotherapy to eradicate the plasma cell or B cell clone responsible for the production of the pathologic MIg [7]. Stokes et al. reported a series of 46 patients with LCPT (21 with MGRS) [8] in whom treatment was associated with a higher rate of improvement in renal function compared to no treatment (32% versus 14%). ESRD occurred in 29% of untreated patients but in none of the treated patients. In our case, the patient achieved partial remission with two cycles of CyBorD regimen. However, the serum free light chain fluctuated after the following two cycles of CyBorD, so daratumumab was added, leading to very good partial remission with improved tubular function and stable kidney function. Daratumumab is an anti-CD38 monoclonal antibody which has been widely used in multiple myeloma and light chain (AL) amyloidosis with good hematologic and organ response [9][S1]. However, the data on daratumumab in non-amyloid MGRS patients are limited. Kastritis et al. summarized their experience using daratumumab-based therapy in 25 MGRS patients (including 22 patients with monoclonal immunoglobulin deposition disease, two patients with C3 nephropathy, and one with proliferative glomerulonephritis with monoclonal immunoglobulin deposits) [S2]. The median follow-up of the cohort was 14 months. The best hematologic response in the involved patients was 74%, with complete response in five (22%), very good partial remission in five (22%), and partial remission in seven (30%) patients. Of note, in our case we used four doses of daratumumab, considering the patient’s financial status and susceptibility to infection. We referred to the protocol of Kastritis et al., applying the short course (1 month–four doses) of daratumumab consolidation strategy after eight cycles of bortezomib, cyclophosphamide, and dexamethasone in 25 patients with AL amyloidosis or light chain deposition disease [S3]. Their experience showed that this short daratumumab consolidation strategy induced complete hematologic response in 32% of patients and was also associated with excellent long-term outcomes. Daratumumab can be considered an effective treatment option for patients with MGRS.

### Which lessons can be drawn from this report?

We describe a rare case of a patient with MGRS characterized by combined LCPT and CSH who achieved VGPR to bortezomib-based chemotherapy combined with a short course of daratumumab. MGRS is an under-recognized disease, and appropriate treatment may delay hematologic and kidney disease progression.

## Supplementary Information

Below is the link to the electronic supplementary material.

Supplementary file1 (DOCX 25 KB)

## Data Availability

All data generated or analysed during this study are included in this published article and its supplementary information files.
