# Supplementary material for: Lessons for the clinical nephrologist: a rare case with MGRS characterized by combined crystalline light chain proximal tubulopathy and crystal-storing histiocytosis responding to daratumumab
Source: J Nephrol. 2023 Mar 1;36(4):1203–7. doi: 10.1007/s40620-023-01584-1 (PMC10227152; doi:10.1007/s40620-023-01584-1)
Supplement: Supplementary file 1 — Supplementary file1 (DOCX 25 KB) [file 40620_2023_1584_MOESM1_ESM.docx]

Supplemental table 1: Previous reports of cases with combined LCPT and CSH

| **Author[Ref]** | **Age/sex** | **Clinical renal manifestation** | **Plasma cell dyscrasia** | **Crystal distribution** | **Treatment** | **Prognosis** |
| --- | --- | --- | --- | --- | --- | --- |
|  |  |  |  |  |  |  |
| Carstens *et al*. [S4] | 57/M | SCr 3.2mg/dl, proteinuria 2 g/d | IgG-κ MM | Proximal TEC, histiocytes, visceral epithelial cells, MC, endothelial cells and BM | Chemotherapy (cytoxan, carmustine, and prednisone) | Die of cardiac arrest, SCr 3.9 mg/ dL |
| Yamamoto *et al*. [S5] | 75/M | SCr 110→630 μmol/L (5 years), proteinuria | IgG-κ MM | Podocytes, PEC, proximal and distal TEC, histiocytes, cornea, myeloma cell, choroid plexus | NA | Die of severe leukocytopenia and thrombocytopenia |
| Kowalewska *et al*. [S6] | 52/F | SCr 1.8→2.0mg/dl, proteinuria 1.3→5 g/d | IgG-κ MM | Podocytes, PEC, proximal and distal TEC, interstitial histiocytes | NA | NA |
| Papla *et al*. [S7] | 51/M | Bence–Jones proteinuria  1.54 g/L | IgG-κ MM | Podocytes, GEC, MC, TEC, interstitial histiocytes, MC, hepatocytes and macrophages in liver, alveoli in resected lung | Chemotherapy deferred  due to lung carcinoma  surgery | Died due to multi- organ failure shortly after lung surgery |
| Tomioka *et al*. [S8] | 46/F | Renal dysfunction | IgG-κ MM | Podocytes, TECs, interstitial histiocytes | Chemotherapy followed  by HCT | SCr dropped |
| Stokes *et al*. [S9] | 41/M | SCr 4.2mg/dl, proteinuria 20.2 g/d, partial Fanconi’s syndrome | IgD-κ MM | Proximal TEC, histiocytes | chemotherapy, and HCT | SCr 2.2mg/dl, Fanconi’s syndrome improve |
| Wu *et al*. [S10] | 48/M | SCr 12.6mg/dl, PCR 3.53 g/gCr | κ MM | Proximal TEC, histiocytes | 8 cycles of bortezomib, cyclophosphamide, and dexamethasone, maintenance therapy with thalidomid | Dialysis |
| Boudhabhay *et al*.[S11] | 60/M | SCr 7mg/dl, albumin 23.8 g/L, proteinuria 6.02 g/d | IgG-κ MM | Proximal TEC, histiocytes, podocytes, MC, anterior stromal cornea and retina | 3 cycles of bortezomib, cyclophosphamide, and dexamethasone, 5 cycles of bortezomib, lenalidomide, and dexamethasone | Dialysis, partial hematologic response |
| Ito *et al*. [S12] | 65/F | SCr 1.15→1.94mg/dl, proteinuria 1.31 g/gCr | IgG-κ MGUS | proximal TEC, interstitial histiocytes, podocytes | 9 courses of bortezomib and dexamethasone | Renal function improved |
| Matthai *et al*. [S13] | 48/F | SCr 6.42mg/dl, proteinuria 4.6 g/d | MM | Podocytes, proximal TEC, histiocytes | NA | NA |
| Nakamura *et al*. [S14] | 66/F | SCr 3.32mg/dl, proteinuria 2.2 g/d | κ-MGRS | proximal TEC, interstitial histiocytes, podocytes | NA | NA |
| Present case | 67/M | SCr 925→160.4μmol/L (10.5→1.8 mg/dl), proteinuria 2.01 g/d | κ-MGRS | Proximal TEC, histiocytes, BM | 4 cycles of CyBorD, 1 cycle of BorD combined with daratumumab and one more cycle of BorD | VGPR, SCr 125-140 μmol/L (1.4→1.6 mg/dl) |

Abbreviation: CSH crystal-storing histiocytosis, BM bone marrow, BorD bortezomib and dexamethasone, CyBorD cyclophosphamide, bortezomib, and dexamethasone, DF dexamethasone, GEC glomerular endothelial cell, HCT autologous hematopoietic cell transplantation, LCPT light chain proximal tubulopathy, MC mesangial cell, MGUS monoclonal gammopathy of undetermined significance, MM multiple myeloma, NA not available, PEC parietal epithelial cell, SCr serum creatinine, TEC tubular epithelial cell, PCR protein/creatinine ratio, VGPR very good partial response

**Referrences:**

S1. Kastritis E, Palladini G, Minnema MC, Wechalekar AD, Jaccard A, Lee HC, et al. Daratumumab-Based Treatment for Immunoglobulin Light-Chain Amyloidosis. N Engl J Med. 2021;385(1):46-58. <https://doi.org/10.1056/NEJMoa2028631>

S2. Kastritis E, Theodorakakou F, Roussou M, Psimenou E, Gakiopoulou C, Marinaki S, et al. Daratumumab-based therapy for patients with monoclonal gammopathy of renal significance. British journal of haematology. 2021;193(1):113-8. <https://doi.org/10.1111/bjh.17052>

S3. Kastritis E, Rousakis P, Kostopoulos IV, Gavriatopoulou M, Theodorakakou F, Fotiou D, et al. Consolidation with a short course of daratumumab in patients with AL amyloidosis or light chain deposition disease. Amyloid : the international journal of experimental and clinical investigation : the official journal of the International Society of Amyloidosis. 2021;28(4):259-66. <https://doi.org/10.1080/13506129.2021.1971192>

S4. Carstens PH, Woo D. Crystalline glomerular inclusions in multiple myeloma. American journal of kidney diseases : the official journal of the National Kidney Foundation. 1989;14(1):56-60. <https://doi.org/10.1016/s0272-6386(89)80095-2>

S5. Yamamoto T, Hishida A, Honda N, Ito I, Shirasawa H, Nagase M. Crystal-storing histiocytosis and crystalline tissue deposition in multiple myeloma. Archives of pathology & laboratory medicine. 1991;115(4):351-4.

S6. Kowalewska J, Tomford RC, Alpers CE. Crystals in podocytes: an unusual manifestation of systemic disease. American journal of kidney diseases : the official journal of the National Kidney Foundation. 2003;42(3):605-11. <https://doi.org/10.1016/s0272-6386(03)00794-7>

S7. Papla B, Spólnik P, Rzenno E, Zduńczyk A, Rudzki Z, Okoń K, et al. Generalized crystal-storing histiocytosis as a presentation of multiple myeloma: a case with a possible pro-aggregation defect in the immunoglobulin heavy chain. Virchows Archiv : an international journal of pathology. 2004;445(1):83-9. <https://doi.org/10.1007/s00428-004-1031-3>

S8. Tomioka M, Ueki K, Nakahashi H, Isoda A, Kuroiwa T, Kaneko Y, et al. Widespread crystalline inclusions affecting podocytes, tubular cells and interstitial histiocytes in the myeloma kidney. Clinical nephrology. 2004;62(3):229-33. <https://doi.org/10.5414/cnp62229>

S9. Stokes MB, Aronoff B, Siegel D, D'Agati VD. Dysproteinemia-related nephropathy associated with crystal-storing histiocytosis. Kidney international. 2006;70(3):597-602. <https://doi.org/10.1038/sj.ki.5001524>

S10. Wu CK, Yang AH, Lai HC, Lin BS. Combined proximal tubulopathy, crystal-storing histiocytosis, and cast nephropathy in a patient with light chain multiple myeloma. BMC nephrology. 2017;18(1):170. <https://doi.org/10.1186/s12882-017-0584-8>

S11. Boudhabhay I, Titah C, Talbot A, Harel S, Verine J, Touchard G, et al. Multiple myeloma with crystal-storing histiocytosis, crystalline podocytopathy, and light chain proximal tubulopathy, revealed by retinal abnormalities: A case report. Medicine. 2018;97(52):e13638. <https://doi.org/10.1097/md.0000000000013638>

S12. Ito K, Hara S, Yamada K, Zoshima T, Mizushima I, Fujii H, et al. A case report of crystalline light chain inclusion-associated kidney disease affecting podocytes but without Fanconi syndrome: A clonal analysis of pathological monoclonal light chain. Medicine. 2019;98(5):e13915. <https://doi.org/10.1097/md.0000000000013915>

S13. Matthai SM, Alexander S, Jacob S, Duhli N, David VG, Varughese S. Crystals, crystals everywhere but not a clue till late… Light chain crystalline proximal tubulopathy with concomitant myeloma cast nephropathy. Indian journal of pathology & microbiology. 2020;63(3):463-6. <https://doi.org/10.4103/ijpm.Ijpm_756_18>

S14. Nakamura Y, Kitamura H, Ikai H, Yamamoto M, Murai Y, Watanabe T, et al. Combined light chain crystalline tubulopathy, podocytopathy, and histiocytosis associated with Bence-Jones κ protein diagnosed via immuno-electron microscopy. CEN case reports. 2021;10(3):453-8. <https://doi.org/10.1007/s13730-021-00588-9>
